# *Baccharis dracunculifolia* DC (Asteraceae) Root Extract and Its Triterpene Baccharis Oxide Display Topical Anti-Inflammatory Effects on Different Mice Ear Edema Models

**DOI:** 10.1155/2023/9923941

**Published:** 2023-05-25

**Authors:** Everton Allan Ferreira, Lucas Sales Queiroz, Gabriella de Faria Silva Facchini, Maria Clara Machado Resende Guedes, Gilson Costa Macedo, Orlando Vieira de Sousa, Ademar A. Da Silva Filho

**Affiliations:** ^1^Faculty of Pharmacy, Department of Pharmaceutical Sciences, Federal University of Juiz de Fora, Juiz de Fora, MG 36036-900, Brazil; ^2^Department of Parasitology, Microbiology and Immunology, Federal University of Juiz de Fora, R. José Lourenço Kelmer s/n, Campus Universitário, 36036-900 Juiz de Fora, MG, Brazil

## Abstract

*B. dracunculifolia* is popularly used to treat skin diseases. This work aimed to evaluate the topical anti-inflammatory properties of *B. dracunculifolia* root extract (BdR) and its major compound baccharis oxide (BOx) on mice ear edema models. BdR was analyzed by GC-MS, and BOx was isolated by chromatographic fractionation. Topical anti-inflammatory activities were determined by using the croton oil, capsaicin, histamine, and phenol-induced mouse ear edema models. *N*-acetyl-*β*-*D*- glucosaminidase (NAG) and myeloperoxidase (MPO) activities, as well as NO dosage and histopathological analyses, were also evaluated. Phytochemical analysis of BdR showed BOx as one of the major constituents. BdR and BOx (both at 0.1, 0.5, and 1.0 mg/ear) significantly reduced croton oil, histamine, and phenol-induced ear edema, while only BOx was effective in reducing capsaicin-induced edema. MPO and NAG activities, as well as NO production, were significantly inhibited by BdR and BOx. Histopathological analysis confirmed the topical anti-inflammatory properties of BdR and BOx. Our findings showed that BdR and BOx demonstrated significant topical anti-inflammatory effects in mouse ear edema induced by different agents, suggesting their possible application on skin inflammatory diseases.

## 1. Introduction

Inflammatory skin conditions, like psoriasis and dermatitis, are one of the major causes of disability due to their negative effects in patients, mainly pain and psychological impact [1]. These skin conditions can be occasional (such as redness) or chronic (such as acne, dermatitis, rosacea, and psoriasis) [2]. Contact dermatitis is an inflammatory response that arises due to the contact with physical or chemical agents that cause skin damage and cellular alterations, as well as releasing of proinflammatory mediators [3]. Additionally, skin inflammatory disorders are related to the stimulation of inflammatory pathways with involvement of many chemical mediators, substrates, and enzymes, which may serve as potential targets for novel anti-inflammatory drugs [4]. Nowadays, topical corticosteroids are used in the treatment of cutaneous inflammatory diseases [5]. However, these medications may cause pruritus, telangiectasia, stomach irritation, and ulceration, among other side effects [6]. Therefore, these drawbacks emphasize the need for discovering new and efficient therapeutic options to treat skin disorders [5]. In this regard, natural products, such as plant extracts and their compounds, can be used as an attractive strategy for treating skin inflammatory illnesses [7].

Plants of the genus *Baccharis* are used in South America as anti-inflammatory to treat skin disorders [8]. Among them, *Baccharis dracunculifolia* De Candole (Asteraceae), known as “alecrim do campo,” is a plant popularly used for the treatment of inflammations and skin diseases [9–11]. Also, the economic and commercial interests of *B. dracunculifolia* have increased in the last years, since this species is one of the most important plant sources of the Brazilian green propolis [8]. *B. dracunculifolia* leaf extracts displayed analgesic and anti-inflammatory effects in mice [10], also exhibiting immunomodulatory [12, 13], antimicrobial [14], and hepatoprotective [15] activities. The anti-inflammatory effects of *B. dracunculifolia* leaf extract have been associated with some phenolic acids, mainly artepillin C, as well as flavonoids, such as isosakuranetin [12]. In addition, it was demonstrated an interesting anti-inflammatory effect of *B. dracunculifolia* essential oil on skin inflammation [9]. Additionally, the previous study showed that the *B. dracunculifolia* root extract exhibits *in vitro* immunomodulatory action in murine macrophages [13]. However, the potential of the *B. dracunculifolia* root extract and its compounds as topical anti-inflammatory agents has not yet been explored.

Then, in this study, it was evaluated the topical anti-inflammatory properties of the *B. dracunculifolia* root extract and its major compound baccharis oxide on skin inflammation through mice ear edema models.

## 2. Materials and Methods

### 2.1. Chemicals

Phenol, croton oil, histamine, capsaicin, and dexamethasone were acquired from Sigma-Aldrich^®^ Co. (St. Louis, MO, USA). Acetone, dichloromethane, and ethanol were obtained from Vetec^®^ Química Farm. Ltda (Rio de Janeiro, RJ, Brazil), while xylazine chloride and ketamine chloride were from Syntec^®^ (Hortolândia, SP, Brazil), and dexchlorpheniramine maleate was from EMS^®^ (Hortolândia, SP, Brazil). All other solvents and chemicals were of analytical grade.

### 2.2. Preparation of the Crude Plant Extract

This research was registered (Number #AE32DB3) at SisGen, the National System for the Management of Genetic Heritage and Associated Traditional Knowledge. After plant authentication (Dr P.L. Viana; voucher specimen CESJ 47482), *B. dracunculifolia* roots were collected in August 2017 at the campus of the Federal University of Juiz de Fora (UFJF). Roots (2300 g) were dried, pulverized, and extracted, by maceration, employing *n*-hexane (5.6 L) as solvent. Next, *n*-hexane was removed via rotary evaporation to yield the *n*-hexane extract of *B. dracunculifolia* roots (BdR, 18.5 g).

### 2.3. Isolation of Baccharis Oxide (BOx)

BdR (10 g) was chromatographed in a vacuum liquid chromatography system using silica gel (Merck, 40–63 mesh) as the stationary phase and combinations of hexane: ethyl acetate as solvents, furnishing 7 fractions: Fr1 (0.30 g, hexane 100%), Fr 2 (1.2 g, hexane: ethyl acetate 85 : 15 v/v), Fr 3 (0.87 g, hexane: ethyl acetate 70 : 30 v/v), Fr 4 (0.90 g, hexane: ethyl acetate 60 : 40 v/v), Fr 5 (0.99 g, hexane: ethyl acetate 50 : 50 v/v), Fr 6 (0.79 g, hexane: ethyl acetate 30 : 70 v/v), and Fr 7 (0.81 g, ethyl acetate 100%). The resulting fraction Fr 2 (1.20 g) was subjected to chromatography in a column using silica gel and acetonitrile 100% as the mobile phase, affording baccharis oxide (BOx, 0.2 g). Chemical structure of BOx was established by GC-MS analysis, ^13^C, ^1^H NMR, and comparison with the literature [16].

### 2.4. GC-MS Analysis

BdR and its isolated compound BOx were analyzed by GC-MS (Shimadzu QP2010 Plus with a Restek RTX-5MS column). Temperature was programed to increase from 120 to 240°C (4°C/min) with an injector temperature of 150°C. Also, an ion-source temperature of 280°C with He (1.0 mL/min) as carrier gas, a split ratio of 1 : 10, and an injection volume of 8 *μ*L were used. The electron ionization mode (70 eV) was used, and the spectra were carried with a scan interval of 0.5 s over the mass range of 40–600 Da. Identification of BdR compounds was achieved by a comparison of their mass spectral fragmentation patterns with those of spectral libraries (NIST 08 and Wiley 7), as well as by their retention indices obtained with reference to a homologous series of *n*-alkanes (C_7_–C_30_) [17].

### 2.5. Animals

**Figure 1:**
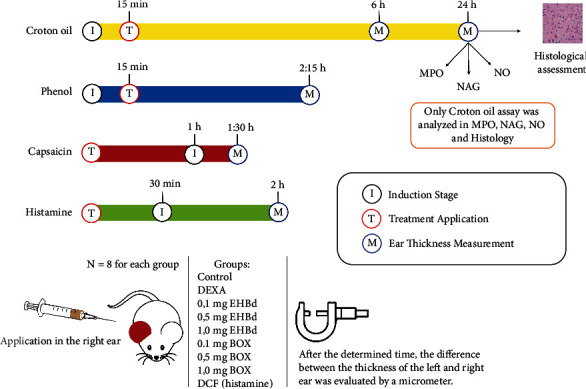
Experimental biological design used in this study.

Male Swiss mice (30–40 g) were kept in groups of eight animals (*n* = 8) and maintained in plastic cages (47 × 34 × 18 cm) under a 12/12 h light-dark cycle, in controlled temperature (22 ± 2°C), with food and water *ad libitum*. Mice were acclimated in the experimentation room for 24 h before the experiments. The euthanasia of the animals was performed with anesthetic overdose (300 mg/kg ketamine and 30 mg/kg xylazine). All experimental procedures were approved by the Ethics Committee on Animal Use/UFJF and conducted in accordance with the guidelines of the COBEA (Brazilian College of Animal Experimentation-Protocol no 022/2018, approved in 08/21/2018). All experiments were performed according to the experimental design (Figure 1).

### 2.6. Croton Oil-Induced Ear Edema Model

Ear edema was produced with croton oil (2.5% v/v in acetone, 20 *µ*L), which was applied on the surface of the right ear, while the left ear received only acetone (20 *µ*L) [7, 18]. The right ear was treated with BdR (0.1, 0.5 and 1.0 mg/ear), BOx (0.1, 0.5 and 1.0 mg/ear), dexamethasone (0.1 mg/ear), and 0.9% NaCl (negative control group). The ear thickness (*µ*m) was measured after 6 and 24 h [7, 9], using a digital micrometer, to evaluate edema development. Edema was measured by the difference in thickness (*µ*m) between the right and left ears [7, 9, 18]. Ear fragments were also used in histopathological analysis and for determination of inflammatory markers.

### 2.7. *N*-Acetyl-*β*-*D*-Glucosaminidase (NAG) and Myeloperoxidase (MPO) Assays

Ear fragments (6 mm circles of tissue) obtained from the croton oil assay (after 24 h) [7, 9, 19] were frozen and subjects to crushing in mortar with 1 mL of HTBA 0.5% in sodium phosphate buffer (80 mM, pH 5.4). After, 1 mL of sodium phosphate buffer was added, and the homogenate was transferred to test tubes and subjected to ultrasound (Unique^®^ 1600) for 10 min. Then, the tubes were centrifuged (3000 RPM, 10 min), and the supernatants were collected for measuring *N*-acetyl-*β*-*D*-glucosaminidase (NAG) and myeloperoxidase (MPO) activities.

To assess MPO activity [7, 9, 19], quadruplicates of 70 *μ*L of the supernatant were placed on a 96-well plate in which were added 3.3′5.5′-tetramethylbenzidine (35 *µ*L, 1.6 mM in DMSO) and H_2_O_2_ (0.0003% v/v, 105 *µ*L diluted in 80 mM sodium phosphate buffer, pH 5.4) into each well. After incubation of microplates (37°C, 5 min), the reaction was stopped by addition of H_2_SO_4_ (4 M, 140 *μ*L). Absorbances were measured (450 nm) in a microplate reader (Thermoplate^®^). Results were expressed as optical density/mg of protein (mOD/mg of protein) [7, 9, 19].

To assess NAG activity [9, 20], quadruplicates of 100 *μ*L of the supernatant were placed on a 96-well plate in which were added NAG (200 *µ*L of 2.24 mM in citrate buffer 0.1 M, pH 4.5) and 50 *µ*L of *p*-nitrophenyl-*N*-acetyl-*β*-*D*-glucosamine. After incubation of microplates (37°C, 10 min), the reaction was stopped by addition of glycine buffer (30 *µ*L of 0.2 M, pH 10.6). A microplate reader, at 405 nm, was used to determine the colorimetric enzymatic activity. Results were expressed as optical density/mg of protein (mOD/mg of protein) [9, 20].

### 2.8. Nitric Oxide Assay

Nitrite, the stable product of nitric oxide (NO), was measured [21]. In this assay, the homogenate was prepared from three ear fragments obtained from the croton oil-induced model. Then, PBS (3 mL, pH 7.4) was added to each ear fragment, which was crushed for 1 min. After, the obtained homogenate was transferred to test tubes and centrifuged (7000 RPM, 15 min). Next, 150 *µ*L of each supernatant was mixed with 150 *µ*L of Griess reagent, and microplates were incubated at room temperature for 30 min. The measurements of absorbance were made in a microplate reader at 540 nm. Nitrite levels were performed in triplicate using a standard curve of nitrite (NaNO_2_) prepared in PBS (pH 7.4). Results were expressed in *µ*M, and experiments were made in triplicate [21].

### 2.9. Histological Assessment

According to the previous methodology [7, 9, 22], ear fragments (6 mm) were preserved in 70% ethanol and 10% formaldehyde (v/v). After, ear fragments were blocked in paraffin, sectioned with a microtome, and stained with hematoxylin-eosin for microscopic analysis. A representative area was selected for qualitative microscopic analysis of the inflammatory tissue (200x magnification) [7, 9].

### 2.10. Phenol-Induced Ear Edema Model

Phenol (10% in acetone, 20 *µ*L) was applied in the right ears of mice, while in the left ears, only acetone was applied [22, 23]. After 15 min, BdR and BOx (both at 0.1, 0.5, and 1.0 mg/ear), as well as dexamethasone (0.1 mg/ear, positive control), were dissolved in acetone and topically administered (20 *µ*L) to the right ears. Saline (20 *µ*L) was used as a negative control. The thickness (*µ*m) and weight (mg) were evaluated after 2 h of treatment, and edema was determined by the difference between the right and left ears [22, 23].

### 2.11. Capsaicin-Induced Ear Edema Model

BdR (0.1, 0.5, and 1.0 mg/ear), BOx (0.1, 0.5, and 1.0 mg/ear), and dexamethasone (0.1 mg/ear, used as a positive control) were dissolved in acetone and administered topically (20 *µ*L) to the right ears of mice, also using saline (20 *µ*L) as a negative control [7, 22]. After 1 h, capsaicin (20 *µ*L of 0.01 mg/*µ*L v/v in acetone) was applied to the inner surface of the right ears of mice, while in the left ears, only acetone was received (20 mL). Then, after 30 min [7], the animals were euthanized, and the measure of edema (thickness) was calculated by the difference between the right and left ears [7, 22].

### 2.12. Histamine-Induced Ear Edema Model

BdR (0.1, 0.5 and 1.0 mg/ear), BOx (0.1, 0.5 and 1.0 mg/ear), and dexchlorpheniramine (0.1 mg/ear) were dissolved in acetone and topically administered (20 *µ*L) to the right ears of mice, using saline (20 *μ*L) as a negative control [24]. After 30 min of treatment, animals were anesthetized (10 mg/kg xylazine and 100 mg/kg ketamine), and histamine (1 *µ*g in 10 *µ*L of saline) was applied intradermally to the right ear, while in the left ear, saline was administered (10 *µ*L). Thickness (mm) of the ears was evaluated after 90 min, and edema was determined by the difference between the right and left ears [24].

### 2.13. Cell Viability

Cell viability of BdR and baccharis oxide (BOx) was determined in RAW264.7 cells (murine macrophage cell line), using the MTT assay [25] and three independent experiments in duplicate.

### 2.14. Statistical Analysis

Differences between groups were assessed by analysis of variance (ANOVA) followed by post-hoc Student-Newman–Keuls using GraphPad Prism^®^ 7.0 program. Data are presented as the mean ± S.E.M. Statistical differences were considered significant at *P* < 0.05.

## 3. Results

### 3.1. GC-MS Analysis

In BdR, 10 compounds were identified by GC-MS (Table 1), including fatty acid derivatives, monoterpenes, and triterpenes. Baccharis oxide (BOx) was identified as one of the major constituents (26.35%) of BdR.

### 3.2. Isolation and Identification of Baccharis Oxide

**Figure 2:**
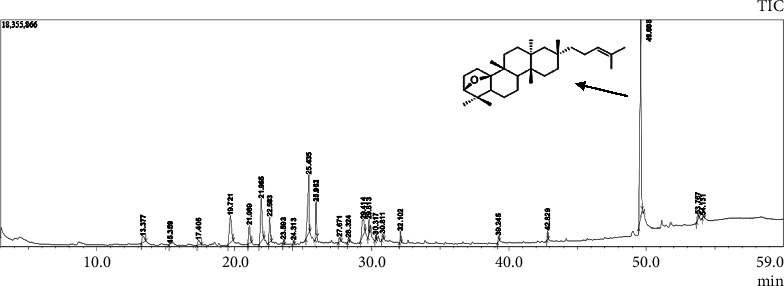
GC-MS chromatogram of BdR and chemical structure of baccharis oxide (BOx, peak at 49.6 min).

BOx (Figure 2) was obtained from BdR followed by few steps of chromatographic fractionation. GC-MS analysis (Figure S1 in supplementary information) and ^13^C NMR (Figure S2 in supplementary information) estimated the purity of BOx to be greater than 95%. Using GC-MS (Figure S1 in supplementary information), as well as ^13^C and ^1^H NMR data analysis (Figures S2-S3 in supplementary information), the identification of BOx was confirmed in comparison to the literature [16].

### 3.3. Anti-Inflammatory Effects of BdR and BOx on Edema Induced by Croton Oil

**Figure 3:**
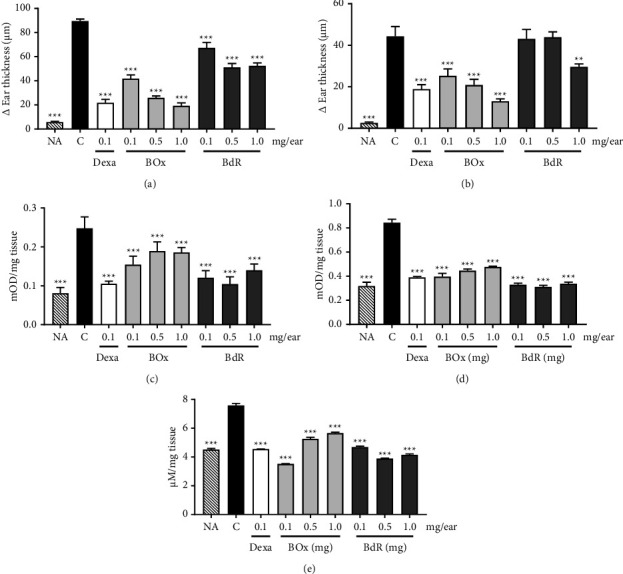
Effect of BdR and BOx on croton oil-induced mouse ear edema, MPO, NAG activity, and NO production. (a) Δ ear edema measured at 6 h and (b) 24 h. (c) MPO activity. (d) NAG activity. (e) NO production. NA, naïve; C, negative control; dexa, dexamethasone; BOx, baccharis oxide; BdR, *B. dracunculifolia* root extract. ^*∗∗∗*^*P* < 0.001 and ^*∗∗*^*P* < 0.01 compared to the control group (ANOVA followed by Student-Newman–Keuls).

As demonstrated in Figures 3(a) and 3(b), the application of croton oil significantly increased ear thickness at both 6 and 24 h after its administration. All tested BdR concentrations reduced croton edema after 6 h, while after 24 h, only at 1.0 mg/mL, BdR significantly reduced edema formation in 33.0%. On the other hand, when BOx was applied topically, it was able to reduce the croton oil edema at all tested doses (0.1, 0.5, and 1.0 mg/mL) after both 6 h and 24 h administration.

At 1.0 mg/ear, BOx exhibited the highest inhibitory activities in ear edema formation of 78.3% ± 2.6 and 70.6% ± 9.0, after 6 and 24 h, respectively, while dexamethasone (at 0.1 mg/ear) reduced the ear thickness in 75.7% ± 3.2 and 57.3% ± 4.9, after 6 and 24 h, respectively. The left ears, which received only vehicle, did not display quantified edema.

Also, the enzymatic activities of MPO and NAG, as well as the activity of NO, were measured in the ears after 24 h of croton oil administration. Topical administration of croton oil on mice ears promoted significant increases in the enzymatic activities of MPO and NAG in comparison with the naive group (Figures 3(c)–3(e)). In contrast, the increase in MPO activity was inhibited by both BdR and BOx at all tested doses. The maximum inhibition of BdR was 57.7% ± 3.7 at 0.5 mg/ear, while BOx showed its maximum inhibition of 37.70% ± 4.3 at 0.1 mg/ear. Similarly, dexamethasone (at 0.1 mg/ear) reduced MPO activity by 57.5% ± 1.3 when compared to the control group (Figure 3(c)).

In addition, both BdR and BOx, at all tested doses, were able to reduce the increase in NAG activity caused by croton oil (Figure 3(d)). At 0.1 mg/ear, BdR and BOx inhibited the NAG activity in 61.1% ± 0.8 and 53.0% ± 1.6, respectively, while dexamethasone (at 0.1 mg/ear) showed an inhibition of 53.8% ± 0.4.

Additionally, as shown in Figure 3(e), croton oil caused an increase in NO production. In contrast, treatment with BdR or BOx reduced NO production at all tested doses, with a maximum inhibition of 48.7% ± 0.2 by BdR (at 0.5 mg/ear) and 53.5% ± 0.2 by BOx (at 0.1 mg/ear), while dexamethasone (at 0.1 mg/ear) reduced NO production by 40.0% ± 0.1 compared to the control group.

**Figure 4:**
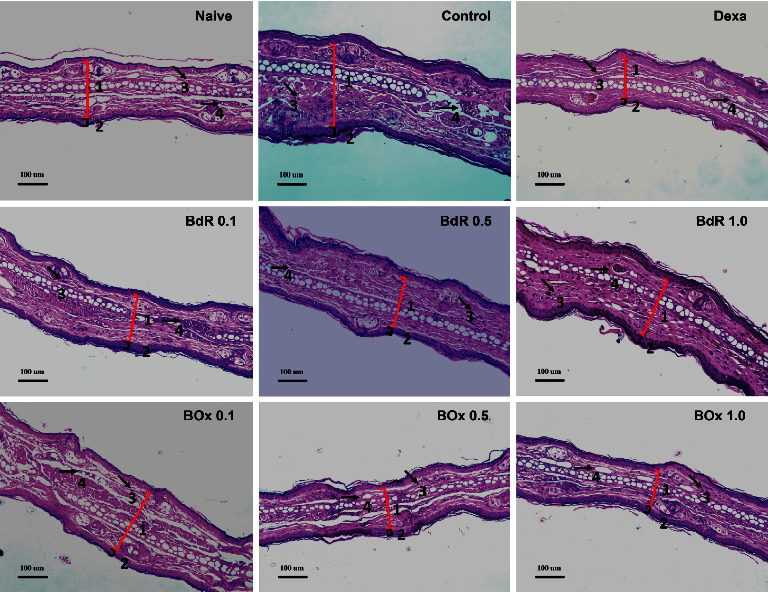
Histological assessment of transverse sections of the ears sensitized with single application of croton oil after 24 h stained with hematoxylin-eosin under qualitative light microscopy (magnification: 200x). Square brackets numbered 1 (red) and 2 indicate ear thickness (dermis and epidermis increase, respectively), and arrow numbered 3 (right down) and 4 (right) indicate inflammatory cells (infiltration of mononuclear leukocytes) and blood vessel (vasodilatation), respectively. The bar represents 100 *µ*m.

As evidenced by ear histopathological analysis (Figure 4), croton oil promoted typical inflammatory effects associated with vasodilatation, edema, marked infiltration of inflammatory cells, and ear thickness (Figure S4 in supplementary information) when compared with the noninflamed ears in the naive groups. The treatments with both BdR (1.0 mg/ear) and BOx (1.0 mg/ear) (Figure 4) promoted reduction in vasodilation, thickness, and cell infiltration induced by croton oil in comparison with the control group. Histopathological analysis also showed that dexamethasone inhibited edema (Figure 4).

### 3.4. Effects of BdR and BOx on Capsaicin- and Phenol-Induced Ear Edema

**Figure 5:**
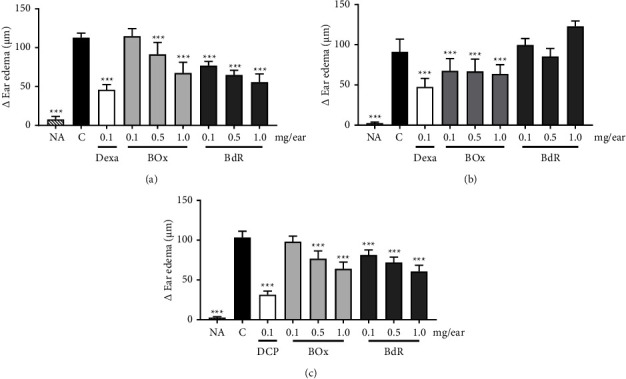
Effect of BdR and BOx on phenol, capsaicin, and histamine-induced mouse ear edema. (a) Phenol-induced edema, (b) capsaicin-induced edema, and (c) histamine-induced edema. NA, naive; C, negative control; Dexa, dexamethasone; DCP, dexchlorpheniramine; BOx, baccharis oxide; BdR, *B. dracunculifolia* root extract. ^*∗∗∗*^*P* < 0.001 compared with the control group (ANOVA followed by Student-Newman–Keuls).

BdR exhibited significant edema thickness reductions on the sensitized ears with phenol (Figure 5(a)), with a maximum inhibition rate of 50.8% ± 3.4 at 1.0 mg/ear, while no edema thickness reduction was observed in the capsaicin model. BOx also reduced phenol edema at 0.5 mg/ear (inhibition of 19.0% ± 4.8) and at 1.0 mg/ear (inhibition of 40.3% ± 4.4). In addition, BOx showed significant reductions of 25.8% ± 5.9, 26.6% ± 6.0, and 30.2% ± 4.5 at 0.1, 0.5, and 1.0 mg/ear, respectively, in capsaicin edema (Figure 5(b)). Dexamethasone was effective in reducing both phenol- and capsaicin-induced edema (Figures 5(a)-5(b)).

### 3.5. Effects of BdR and BOx on Histamine-Induced Ear Edema

In histamine-induced edema, at 1.0 mg/ear, both BdR and BOx caused significant reductions in edema formation of 41.5% ± 2.8 and 38.2% ± 2.9, respectively, when compared to negative control (Figure 5(c)). Dexchlorpheniramine (DCP), used as positive control, reduced histamine-induced edema in 69.8% ± 1.6 (Figure 5(c)).

### 3.6. Cell Viability

**Figure 6:**
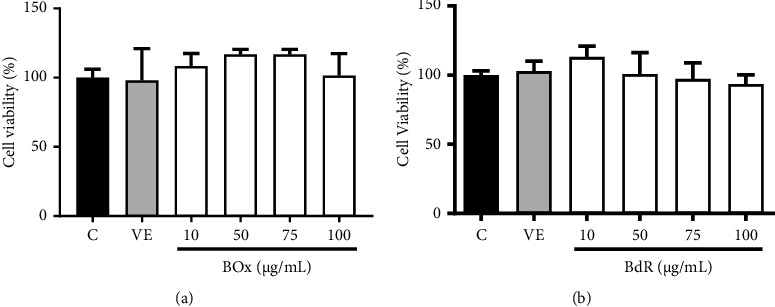
Cell viability of BOx and BdR by MTT assay. (a) Evaluation of the effect of BOx and (b) BdR on the viability of RAW 264.7 cells. C, Control; VE, DMSO 0.5%; BOx; and BdR at 10, 50, 75, and 100 *µ*g/mL.

The MTT assay demonstrated that BdR and BOx are not cytotoxic to the RAW 264.7 cell lines at the tested concentrations (10–100 *µ*g/mL) (Figure 6).

## 4. Discussion

The economic, commercial, and anti-inflammatory potential of *B. dracunculifolia* makes this species a prospective source of bioactive compounds for the development of pharmaceutical products to treat inflammatory illnesses [12]. In this regard, this study assessed the topical anti-inflammatory effects of BdR and its triterpene BOx in mice ear models using different inflammatory agents.

Although few reports have been published about the composition of *B. dracunculifolia* roots, previous phytochemical studies showed the presence of triterpenes, mainly BOx and friedelanol [11, 13]. In our present study, the phytochemical analysis of BdR by GC-MS allowed the identification of BOx as one of the major constituents. Among the identified triterpenes in BdR, friedelin and friedelanol are well known for their anti-inflammatory activity in the cutaneous inflammatory processes [26, 27]. In contrast, there is no previous report about the anti-inflammatory activity of BOx. Because BOx is one of the major constituents and might be related to the activities of BdR, we have isolated it by chromatographic fractionation.

Before topical applications on mice ear edema models, BOx and BdR were *in vitro* evaluated in a MTT assay, showing no cytotoxicity against mammalian cells when tested up to 100 *µ*g/mL.

Then, the anti-inflammatory properties of BdR and BOx were first analyzed on the skin inflammation model using croton oil as phlogistic agent on mice ears. The doses used of BdR and BOx were established in accordance with the previous protocols [7, 22]. The considerable increase in ear thickness and edema formation promoted by croton oil was inhibited by the application of BdR and BOx. Croton oil contains a series of phorbol-12, 13-diesters, which activates protein kinase C and other inflammatory mediators, promoting irritant and cutaneous signs similar to psoriasis [28, 29]. Also, topical application of croton oil triggers local inflammation, inducing edema and erythema, also increasing vascular permeability, leukocytes infiltration, synthesis of eicosanoids, and liberation of histamine and serotonin [19]. In addition, croton oil induces skin inflammation, activating an enzymatic cascade, including phospholipase A_2_ (PLA_2_), which further induces the releasing of arachidonic acid (AA), prostaglandins, and platelet activation factor [30, 31]. Corticosteroids, such as dexamethasone, present significant anti-inflammatory effects in this croton oil model, as well as COX and 5-LOX inhibitors and leukotriene B4 (LTB4) antagonists [32, 33]. Thus, the inhibition of croton oil inflammation by both BdR and BOX suggests that they may interfere in relevant steps throughout the inflammatory cascade triggered in this model. To evaluate the effects of BdR and BOx in the infiltration of leukocytes, the MPO activity was analyzed in ears samples. MPO is an enzyme found in intracellular granules of neutrophils [34] that are an indicator of the leucocyte chemiotaxis process and a marker of the presence of polymorphonuclear cells in the inflammatory exudate [35]. Based on results of MPO activity, BdR and BOx produced important inhibitions of cell migration and neutrophil accumulation, which may avoid an amplification of the inflammatory process [3].

Additionally, the infiltration of mononuclear cells in ears after administration of croton oil was also assessed by NAG activity, since NAG is an enzyme produced by activated macrophages [36]. Although the elevation of NAG release by mononuclear cells is typical of chronic inflammation, acute skin inflammation also may evidence an increment of NAG activity [36]. In the same manner of MPO activity, the treatment with BdR and BOx also promoted a decrease in NAG activity. Also, it was observed that both BdR and BOx decrease NO concentration after croton oil exposure. NO is a mediator involved principally in vascular homeostasis, and in excess, NO can initiate several inflammatory diseases/processes, such as septic shock, psoriasis, and systemic lupus [37, 38].

Additionally, histological analyses were consistent with the inhibitory effects of BdR and BOx on cellular infiltration and on ear edema caused by croton oil. Histological analysis of the ears, exposed with croton oil and treated with BdR and BOx, showed infiltration of inflammatory cells similar to dexamethasone-treated ears. Also, BdR and BOx reduced all evaluated inflammatory parameters, such as monocytes/macrophages and neutrophils migration, as well as edema and epidermis thickness. Taken together, these data suggested that both BdR and BOx may decrease the inflammatory response, inhibiting edema and chemotaxis of inflammatory cells and decreasing vascular permeability in croton oil edema.

Moreover, in the phenol-induced edema model, the direct contact of phenol with the skin may cause rupture of the keratinocyte membranes, resulting in releasing of many cytokines, such as IL-8, IL-1*α*, and TNF-*α*, which leads to the release of other inflammatory mediators, like AA metabolites and ROS [39]. Then, phenol is considered a suitable inflammatory agent for simulating dermatitis in mice [40, 41]. In this model, both BdR and BOx demonstrated significant reduction in ear edema, with noticeable potential in the treatment of contact dermatitis, suggesting that their anti-inflammatory activities may also be related to a possible decrease in the production of AA metabolites and/or with some antioxidant likely properties, which should be further explored in the future.

Additionally, capsaicin is an alkaloid found in hot peppers (*Capsicum* sp), which in contact with epidermis activates vanilloid receptor subtype 1 (TRPV1), elicits a quick response via the release of neuropeptides (such as substance P and tachykinins), and monoamines (serotonin and histamine) [32]. Dexamethasone and antihistamines present significant antiedematous effect in this model [32]. Interestingly, BOx was able to reduce edema on the capsaicin model, while the topical application of BdR did not present any significant decrease, suggesting that BOx may also interfere in mediators or receptors involved in capsaicin-activated inflammatory pathways.

Histamine, an amine released by activated mast cells, increases vasodilation and vascular permeability and is involved in the pathogenesis of many allergic diseases, such as allergic asthma, atopic dermatitis, and allergic rhinitis [24, 32]. Intriguingly, our findings demonstrated that both BdR and BOx reduce histamine-induced ear edema, suggesting that BdR and BOx may also interfere with histamine release.

Furthermore, considering the anti-inflammatory effects observed by BdR, although a significant anti-inflammatory contribution of BOx is present, we cannot discard the effects of other active compounds in the extract, such as friedelin and friedelanol. In this regard, previous studies have shown that friedelin possess anti-inflammatory properties [42], while friedelanol inhibits NO production by murine macrophage RAW264.7 cell [26]. Thus, the topical anti-inflammatory effects of BdR may be due to not only to BOx but also to a combined effect of all its active compounds, resulting in a final and effective anti-inflammatory action. Finally, BdR and BOx may act at different points of inflammation, which may contribute to their topical anti-inflammatory efficacy. Although the precise anti-inflammatory mechanisms of BOx and BdR should be further clarified, BOx presents comparable topical effectiveness to corticosteroid dexamethasone in reversing the inflammatory process of croton ear edema.

## 5. Conclusions

This work evidenced, for the first time, that *B. dracunculifolia* root extract (BdR) and baccharis oxide (BOx) demonstrated significant anti-inflammatory effects in ear edema caused by different agents, suggesting their possible application on inflammatory skin conditions, such as psoriasis and dermatitis. Finally, although remarkable anti-inflammatory effects were observed, the precise mechanisms and safety profiles of BdR and BOx should be further investigated.

## Figures and Tables

**Table 1:** Identified compounds in the BdR by CG-MS analysis.

Compounds	RT	A (%)	RI_exp_	RI_lit_	SI
Spathulenol	15.350	0.19	1580	1577	90
Zierone	21.092	2.79	1693	1694	85
Methyl palmitate	24.317	0.30	1920	1926	96
Palmitic acid	25.433	11.32	1966	1968	94
Ethyl palmitate	25.958	3.30	1980	1978	95
Ethyl linoleate	29.818	1.87	2160	2162	93
Butyl palmitate	30.317	0.67	2180	2188	95
Baccharis oxide	49.608	26.35	^*∗∗*^	2737	88
Friedelin	53.767	1.85	^*∗∗*^	2858	90
Friedelanol	54.133	1.52	^*∗∗*^	2875	90

RT: retention time; A (%): area of each peak relative to the total area of the peaks; RI_exp_: calculated retention index; RI_lit_: retention index from literature; SI: similarity index; ^*∗∗*^: not possible to calculated.

## Data Availability

The data used to support data used to support the findings of this study are included within the article.

## Supplementary Materials

Supplementary Materials The supplementary information consists of the GC-MS, ^13^C, and ^1^H NMR data analysis, as well as additional histological assessment results. Supplementary Figure S1: GC-MS chromatogram and mass spectrum of the isolated compound baccharis oxide (BOx). Supplementary Figure S2: ^13^C NMR data analysis (CDCl_3_, 125 MHz). Supplementary Figure S3: ^1^H NMR data analysis (CDCl_3_, 500 MHz). Supplementary Figure S4: histological assessment of transverse sections of the ear of mice sensitized with single application of croton oil after 24 h stained with hematoxylin-eosin under qualitative light microscopy (magnification: 100x).
