# Supplementary material for: Baccharis dracunculifolia DC (Asteraceae) Root Extract and Its Triterpene Baccharis Oxide Display Topical Anti-Inflammatory Effects on Different Mice Ear Edema Models
Source: Evid Based Complement Alternat Med. 2023 May 25;2023:9923941. doi: 10.1155/2023/9923941 (PMC10234725; doi:10.1155/2023/9923941)
Supplement: Supplementary Materials — The supplementary information consists of the GC-MS, 13C, and 1H NMR data analysis, as well as additional histological assessment results. Supplementary Figure S1: GC-MS chromatogram and mass spectrum of the isolated compound baccharis oxide (BOx). Supplementary Figure S2: 13C NMR data analysis (CDCl3, 125 MHz). Supplementary Figure S3: 1H NMR data analysis (CDCl3, 500 MHz). Supplementary Figure S4: histological assessment of transverse sections of the ear of mice sensitized with single application of croton oil after 24 h stained with hematoxylin-eosin under qualitative light microscopy (magnification: 100x). [file 9923941.f1.zip › Supplementary Figure S4 .docx]

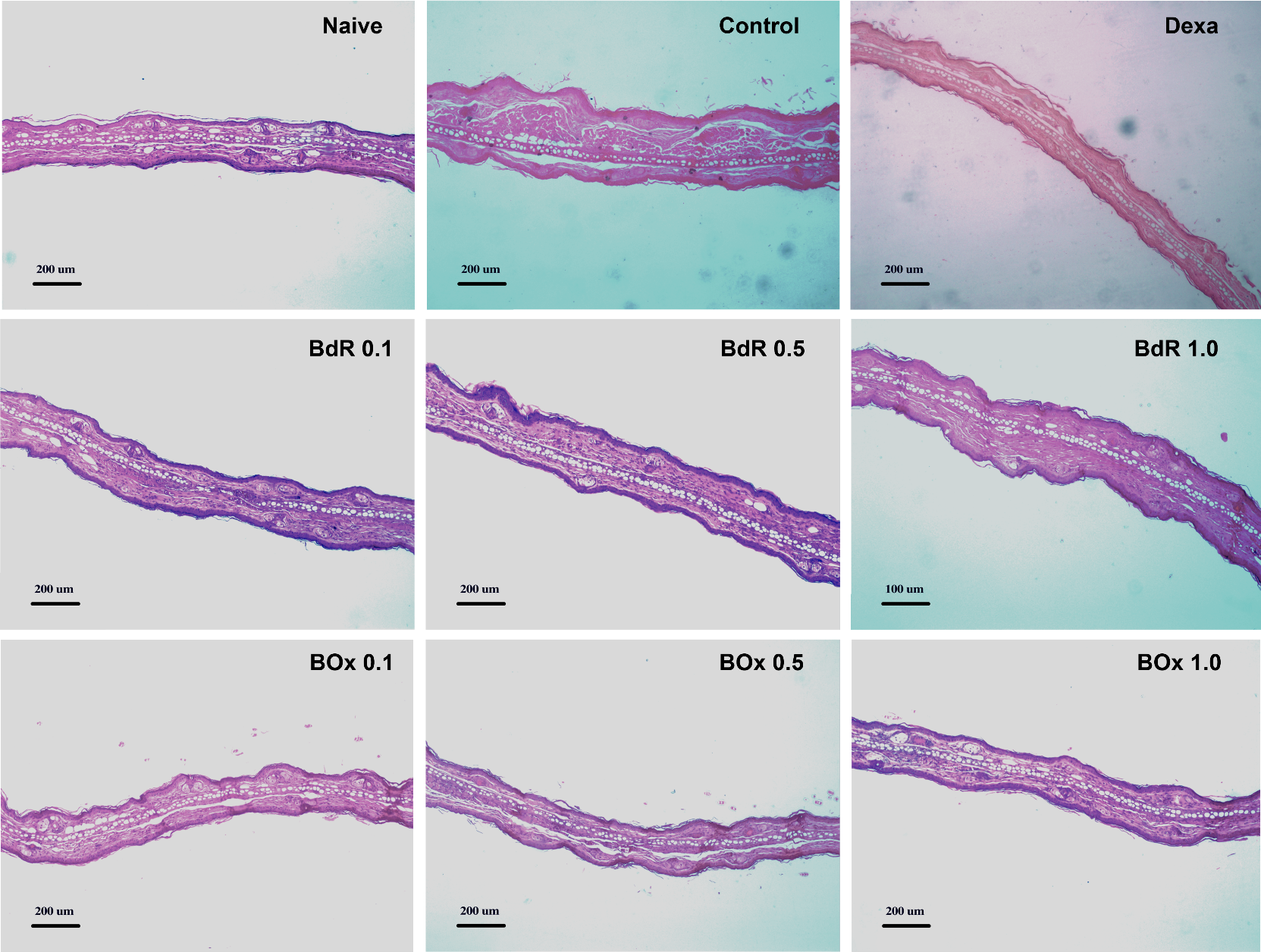


**Supplementary Figure S4** Histological assessment of transverse sections of the ear of mice sensitized with single application of croton oil after 24h stained with hematoxylin-eosin under qualitative light microscopy (magnification: 100x). Bar represents 200 µm.
